# Early real-world outcomes of intravitreal aflibercept 8 mg in treatment-Naïve neovascular AMD: AI-assisted fluid volume analysis

**DOI:** 10.1186/s40942-025-00665-6

**Published:** 2025-04-08

**Authors:** Jennifer Cattaneo, Eva C. De Oliveira Figueiredo, Andrea Montesel, Sandra Vermeirsch, Chiara M. Eandi

**Affiliations:** 1 Fondation Asile des Aveugles, Department of Ophthalmology, Jules-Gonin Eye Hospital, University of Lausanne, Avenue de France 15, Lausanne, 1001 Switzerland; 2 Moorfields Eye Hospital NHS Foundation Trust, London, UK; 3 Department of Surgical Sciences, University of Torino, Torino, Italy

**Keywords:** Aflibercept 8 mg, Anti-VEGF, Age-related macular degeneration, Retina, Optical coherence tomography, Real-world, Early fluid control, Artificial intelligence

## Abstract

**Background:**

This study was conducted as a retrospective, exploratory analysis to assess early anatomical and functional effects of intravitreal aflibercept 8 mg in neovascular age-related macular degeneration (nAMD) in a small cohort of patients.

**Methods:**

This retrospective study was conducted at the Jules Gonin Eye Hospital in Lausanne, Switzerland, and included treatment-naïve patients with nAMD. Patients received a minimum of two intravitreal injections (IVT) of aflibercept 8 mg over a 3-month period. Key outcomes assessed were changes in best-corrected visual acuity (BCVA), central subfield thickness (CST), mean retinal thickness (RT), total fluid (TF) volume which was calculated as the sum of intraretinal fluid (IRF), subretinal fluid (SRF) and pigment epithelial detachment (PED) volumes. These parameters were evaluated at baseline, month 1, and month 3 using the RetinAI Discovery^®^ platform, an artificial intelligence-based analysis system.

**Results:**

10 eyes of 10 patients were enrolled. Mean age was 77.7 ± 12.5 years. Comparative analysis between baseline, month 1, and month 3 revealed statistically significant reduction in CST, RT and TF volume, indicating a positive early response to treatment. One adverse event of intraocular inflammation (IOI) occurred in one patient (10%) after the second IVT injection. Treatment was subsequently interrupted, and IOI resolved with topical corticosteroids therapy.

**Conclusion:**

Intravitreal aflibercept 8 mg demonstrated early anatomical and functional improvements in nAMD treatment-naïve patients after the first 3-months. The use of the AI-based analysis allowed for detailed and automated assessment of retinal changes, providing valuable insights into early treatment effects. Given the retrospective design and small cohort, further studies are warranted to assess long-term outcomes and the potential predictive value of early changes on long-term visual prognosis and safety.

**Clinical trial number:**

Not applicable.

## Background

Neovascular age-related macular degeneration is considered as a major public health concern, as it is one of the leading causes of blindness among the elderly [1]. The advent of anti-VEGF therapies has revolutionized nAMD management and treatment, dramatically improving visual outcomes and enhancing the quality of life for affected individuals [2]. Nowadays, IVT of anti-VEGF agents are the standard of care for treating nAMD [3]. Over the years, various anti-VEGF agents have been employed to manage this condition. Despite considerable advancements, the duration of VEGF suppression varies between different drugs and can differ among individual patients. Consequently, the development of novel therapies continues to target the unmet needs in anti-VEGF treatment. One promising strategy to tackle these challenges involves extending the duration of drug action in the vitreous, thereby improving disease management and reducing the need for frequent treatments [4]. Newer anti-VEGF agents, such as brolucizumab and faricimab, have recently demonstrated visual improvements and fluid control comparable to those achieved with the widely used aflibercept 2 mg [5, 6]. This longer-lasting activity could reduce the treatment burden, and enhance patient compliance, all while maintaining sustained disease control and ensuring a favorable safety profile. Another important approach to tackling these challenges has been increasing the dosage of existing anti-VEGF agents, such as aflibercept, from 2 mg to 8 mg. The rationale for this fourfold increase is to prolong VEGF inhibition, thereby reducing the treatment burden. In the CANDELA trial, the new formulation of aflibercept 8 mg/0.07 mL has shown promises in decreasing injection frequency and extending treatment intervals [7]. Evidence from this clinical trial demonstrated that this higher dose of aflibercept provides durable disease control, with functional and anatomical outcomes comparable to the lower 2 mg dose, while offering the benefit of longer dosing intervals [7]. In January 2024 aflibercept 8 mg, (Eylea™ 8 mg/0,07 ml, Bayer, Germany) was authorized across the European Union and Switzerland for the treatment of nAMD. The approval was based on the 48-week results from the Phase 3 randomized, double-masked, active-controlled pivotal trials (PULSAR and PHOTON) [8, 9]. These studies confirmed that aflibercept 8 mg, administered every 12 or 16 weeks following three initial monthly doses, achieved non-inferior and clinically equivalent vision outcomes compared to the 2 mg dose given every 8 weeks, while maintaining a similar safety profile. These findings suggested that aflibercept 8 mg may significantly reduce injection frequency without compromising efficacy.

In the context of nAMD management, artificial intelligence is now playing an increasingly important role in processing optical coherence tomography (OCT) images, which are crucial for routine management, treatment planning, and follow-up. AI-driven models, particularly those employing deep learning and machine learning algorithms, have demonstrated significant accuracy in quantifying biomarkers such as central retinal thickness, fluid volumes (SRF, IRF), and PED volumes. These innovations enhance the investigation of clinical and topographical features of nAMD, improving disease progression monitoring and the prediction of treatment responses [10, 11].

This study aims to evaluate the early anatomical and functional outcomes of intravitreal aflibercept 8 mg in treatment-naïve nAMD patients in a real-world clinical setting, with a focus on assessing fluid volumes control through an AI algorithm analysis. Due to restricted sample size, this study wants to provide initial insights that will require confirmation in larger, more comprehensive studies.

## Methods

### Study design and population

This is a retrospective, observational, monocentric cohort study, conducted at the Jules Gonin Eye Hospital, Lausanne, Switzerland. We extracted data from the electronic medical records, and we enrolled treatment-naïve patients diagnosed with nAMD from March to September 2024 who received aflibercept 8 mg as a first line treatment. Patients with type 1, type 2, and type 3 MNV, as well as polypoidal choroidal vasculopathy (PCV), were included. All patients received their first injection on the same day as their appointment at the medical retina center.

The study protocol and analysis adhered to the tenets of the Declaration of Helsinki and the research protocol was approved by the local Ethics Committee (CERVD: 2017 − 00493). Enrolled patients signed written informed consent. The eligibility criteria included: (1) patients aged > 55 years; (2) diagnosis of nAMD at baseline; (3) follow up of minimum 12 weeks; (4) naïve eyes treated with at least two intravitreal aflibercept 8 mg injections (8 mg/0.07 mL).

Eyes presenting any of the following conditions were excluded from the study: (1) history of any other chorioretinal disease or optic nerve disorders limiting visual function; (2) significant optic media opacities and/or insufficient fixation to allow high-quality imaging; (3) myopia > 6 diopters (D) of sphere or 3 D of cylinder and/or axial length > 25.5 mm; and (4) recent history of ocular surgical procedures.

### Data collection

Multimodal imaging studies were reviewed by one independent and masked reader (J.C.). In addition to the demographic features and laterality, the following clinical findings were recorded: BCVA using Snellen decimal charts, intraocular pressure (IOP) and the number of aflibercept 8 mg injections during the examined period. All patients underwent a multimodal imaging evaluation that included Scanning Laser Ophthalmoscopy (SLO)-infrared images (IR), structural OCT (Spectralis HRA + OCT; Heidelberg Engineering, Heidelberg, Germany), and at least one ancillary imaging modality between OCT-angiography (Spectralis HRA + OCT), fluorescein angiography (FA) and indocyanine green angiography (ICGA) (Spectralis HRA + OCT). OCT scans were obtained with a volumetric acquisition (20°x20° field, 97 sections, Automatic Real Time Function 12 scans).

### OCT grading

The following OCT biomarkers were graded at each follow-up visit: presence/absence of (1) intraretinal fluid (IRF) (2) subretinal fluid (SRF) (3) subretinal hyperreflective material (SHRM) and (4) PED. The mean value was used for statistical analysis of metric features. For categorical features, in cases of disagreement or uncertainty regarding a single result, a further assessment was conducted by a second experienced senior retina specialist (C.M.E). OCT volumes were analyzedby the Discovery^®^ platform (Discovery OCT Fluid and Biomarker Detector, RetinAI AG, Switzerland) which facilitates the automated segmentation of retinal and choroidal thicknesses, as well as the quantification of retinal volumes and fluid compartments. The segmentation process includes various retinal and choroidal layers, such as the retinal nerve fiber layer (RNFL), the ganglion cell layer and inner plexiform layer (GCL + IPL), the inner nuclear layer and outer plexiform layer (INL + OPL), the outer nuclear layer (ONL), the photoreceptor layer along with the retinal pigment epithelium (PR + RPE), the choriocapillaris and choroidal stroma (CC + CS), the central subfield thickness (CST) (µm) and mean retinal thickness (RT) (µm). Furthermore, the software provides an automated volumetric measurement of IRF volume (nL), SRF volume (nL), PED volume (nL) using the OCT cube scan at each visit. An additional metric, total fluid (TF) volume, was calculated as the sum of IRF, SRF, and PED volumes. We measured the presence of fluids over the whole 6 mm ETDRS macular grid. When an error in automated thickness and/or volumes was present, a manual annotation was performed.

### Statistical analysis

Frequency distributions and descriptive statistics were employed to summarize qualitative variables. Data are represented as mean ± standard deviation (SD). The normality of all data samples was assessed with the Kolmogorov–Smirnov test. Because the use of parametric statistics was not possible, a Wilcoxon signed-rank test was applied to compare longitudinal data over time. Statistical analyses were performed using GraphPad Prism V10.4.0 for Windows (GraphPad Software, Boston, Massachusetts USA). A *p*-value ≤ 0.05 was considered statistically significant.

## Results

### Characteristic of the study population

A total of 10 eyes from 10 patients were included in the analysis. Of these, 5 patients were female (50%), and the mean age of the study population was 77.7 ± 12.5 years (range: 57 to 94 years). The baseline characteristics of the study cohort are summarized in Table 1. A loading phase of three-monthly injections was completed in 5 cases (50%), while in 5 cases (50%) the loading phase was not completed. In 4 eyes, this was due to macular dryness observed after the first injection, leading to subsequent management under a treat-and-extend (T&E) regimen. In one case, intraocular inflammation occurred after the second injection, and the patient was switched to an alternative anti-VEGF therapy.

**Table 1:** Baseline characteristics of the study population

Nr of eyes	10		
Gender % (n)	F = 50% (5)	M = 50% (5)	
Mean age (years ± SD)	77.7 ± 12.57	Min = 57	Max = 94
Mean Nr of IVT ± SD	2.5 ± 0.53	Min = 2	Max = 3
IOI (n)	10% (1)		

SD = standard deviation; F = female; M = male; IVT = intravitreal I njection; IOI = intraocular inflammation

### Functional and anatomical outcomes

Functional and anatomical outcomes at 1 month and 3 months follow-up are presented in Table 2. Mean BCVA of the complete cohort at baseline was 0.54 (± 0.37) Snellen decimals and at 3-month follow up was 0.77 (± 0.48) Snellen decimals. Mean IOP was 12.7 (± 3.0) mmHg at baseline with no pressure spikes during the follow up period. SHRM was present at baseline in 6 eyes (60% of cases) and decreased to 4 eyes (40%) at 3-month follow up. Retinal hemorrhage was present in 2 eyes (20%) at baseline and had completely resolved by the end of the first month.

SRF was present in 9 eyes (90%) at baseline with only 2 eyes (20%) showing SRF at the 3-month follow-up (mean baseline SRF = 194.50 ± 309.60 nL, maximum range: 1014nL; mean 1-month SRF = 6.70 ± 13.65 nL, maximum range: 43 nL; mean 3-month SRF = 9.0 ± 18.15 nL). Similarly, IRF was present in 6 eyes (60%) at baseline (mean baseline IRF fluid = 70.20 ± 140.0 nL) and decreased to 2 eyes (20%) at the 1-month follow up (mean IRF at 1 month = 6.90 ± 16.27 nL). This reduction was maintained at the 3-month follow-up (mean IRF at 3 months = 10.89 ± 21.66 nL). Furthermore, at baseline PEDs were present in 9 eyes with a mean volume of 189.1 ± 262.0 nL. PED volume was significantly decreased (*p* = 0.023) by 1-month follow up and the mean volume was 112.9 ± 121.80 nL, while at 3-month follow up the mean volume was 143.4 ± 171.7 nL.

As reported in Table 3, overall mean RT and CST at baseline were 321.3 ± 33.78 μm and 416.9 ± 157.4 μm respectively, which significantly decreased at 1-month (293.0 ± 21.26 μm, *p* = 0.002 and 329.6 ± 96.21 μm, *p* = 0.0039) and 3-month follow-up (287.6 ± 15.27 μm, *p* = 0.0039 and 320.9 ± 97.99 μm, *p* = 0.0078).

**Table 2:** Functional and anatomical characteristics

	Baseline	1-Month	3-Month
Mean BCVA (Snellen Decimal ± SD and range)	0.54 ± 0.37 Min = 0.1; Max = 1.0	0.66 ± 0.41 Min = 0.1; Max = 1.25	0.77 ± 0.48 Min = 0.1; Max = 1.25
Mean IOP (mmHg ± SD)	12.70 ± 3.02	11.60 ± 2.72	14.11 ± 2.98
PED (n)	90% (9)	90% (9)	70% (7)
SRF (n)	90% (9)	30% (3)	20% (2)
IRF (n)	60% (6)	20% (2)	20% (2)
SHRM (n)	60% (6)	40% (4)	40% (4)
retinal hemorrhage (n)	20% (2)	0% (0)	0% (0)

BCVA = Best corrected visual acuity; ped = pigment epithelium detachment; srf = subretinal fluid; irf = intraretinal fluid; shrm = subretinal hyper-reflective material; sd = standard deviation; iop = intraocular pressure

**Table 3:** Retinal features analysis and their variation over time

	Baseline	1 Month	3 Months
PED (nL ± SD)	189.1 ± 262.0	112.9 ± 121.8	143.4 ± 171.7
SRF (nL ± SD)	194.5 ± 309.6	6.70 ± 13.65	9.0 ± 18.15
IRF (nL ± SD)	70.2 ± 140.0	6.90 ± 16.27	10.89 ± 21.66
TF (nL ± SD)	439.6 ± 434.6	126.8 ± 132.0	132.7 ± 178.0
CST (µm ± SD)	416.9 ± 157.4	329.6 ± 96.21	320.9 ± 97.99
Mean RT (µm ± SD)	321.3 ± 33.78	293.0 ± 21.26	287.6 ± 15.27

PED = Pigment epithelium detachment; SRF = subretinal fluid; IRF = intraretinal fluid; TF = total fluid; CST = central subretinal thickness; RT = retinal thickness; SD = standard deviation

**Fig. 1:**
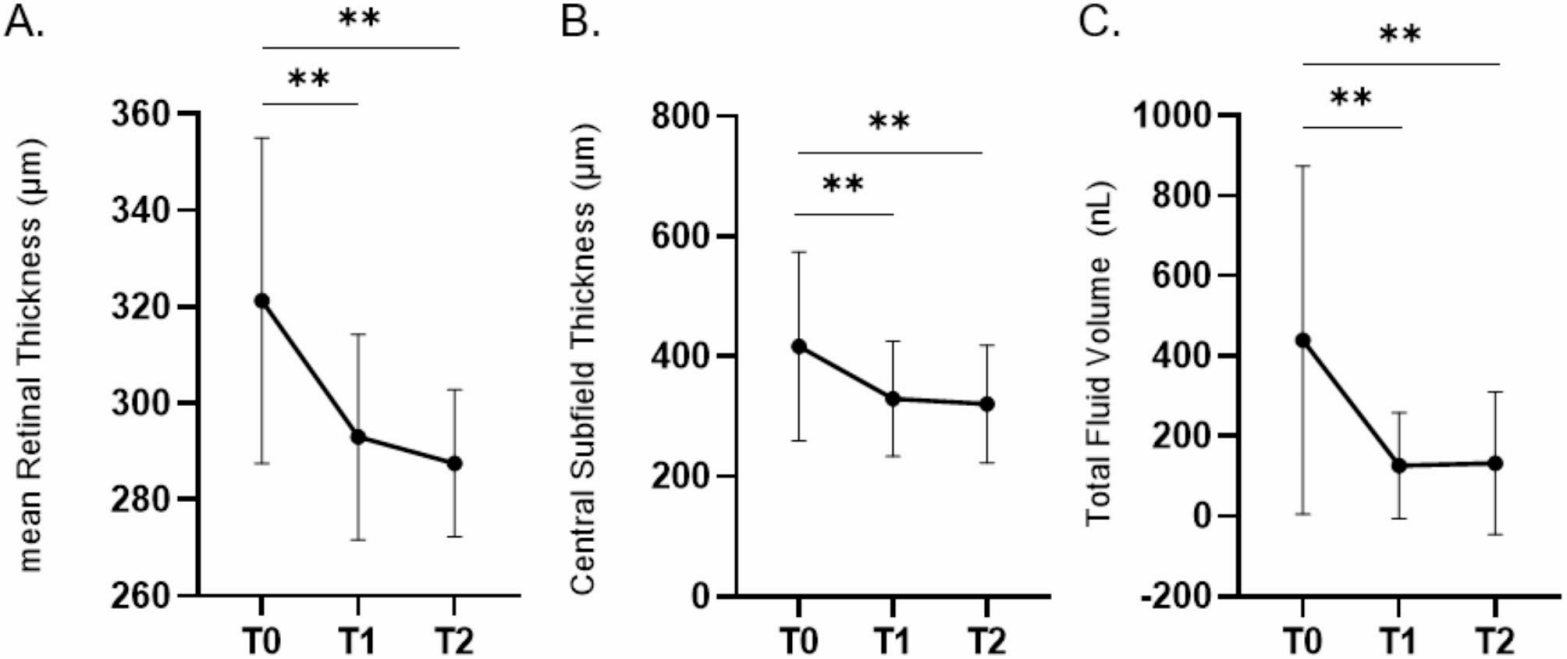
Changes in retinal anatomical features. (**A**) Curve of changes in mean retinal thickness from baseline to 3 months follow-up. After treatment with aflibercept 8 mg mean RT significantly decreased compared to baseline. (**B**) Central Subfield Thickness (CST) changes overtime. (**C**) Fluid status at baseline and during follow-up represented as sum of liquids (intraretinal fluid, subretinal fluid and PED). ** *p* ≤ 0.01. Error bars represent standard deviation (SD) T0 = Baseline; T1 = 1 month; T2 = 3 months

Regarding changes in mean RT, CST and TF volume we observed a trend of early macular dryness as early as the 1-month mark, as shown in Fig. 1.

**Fig. 2:**
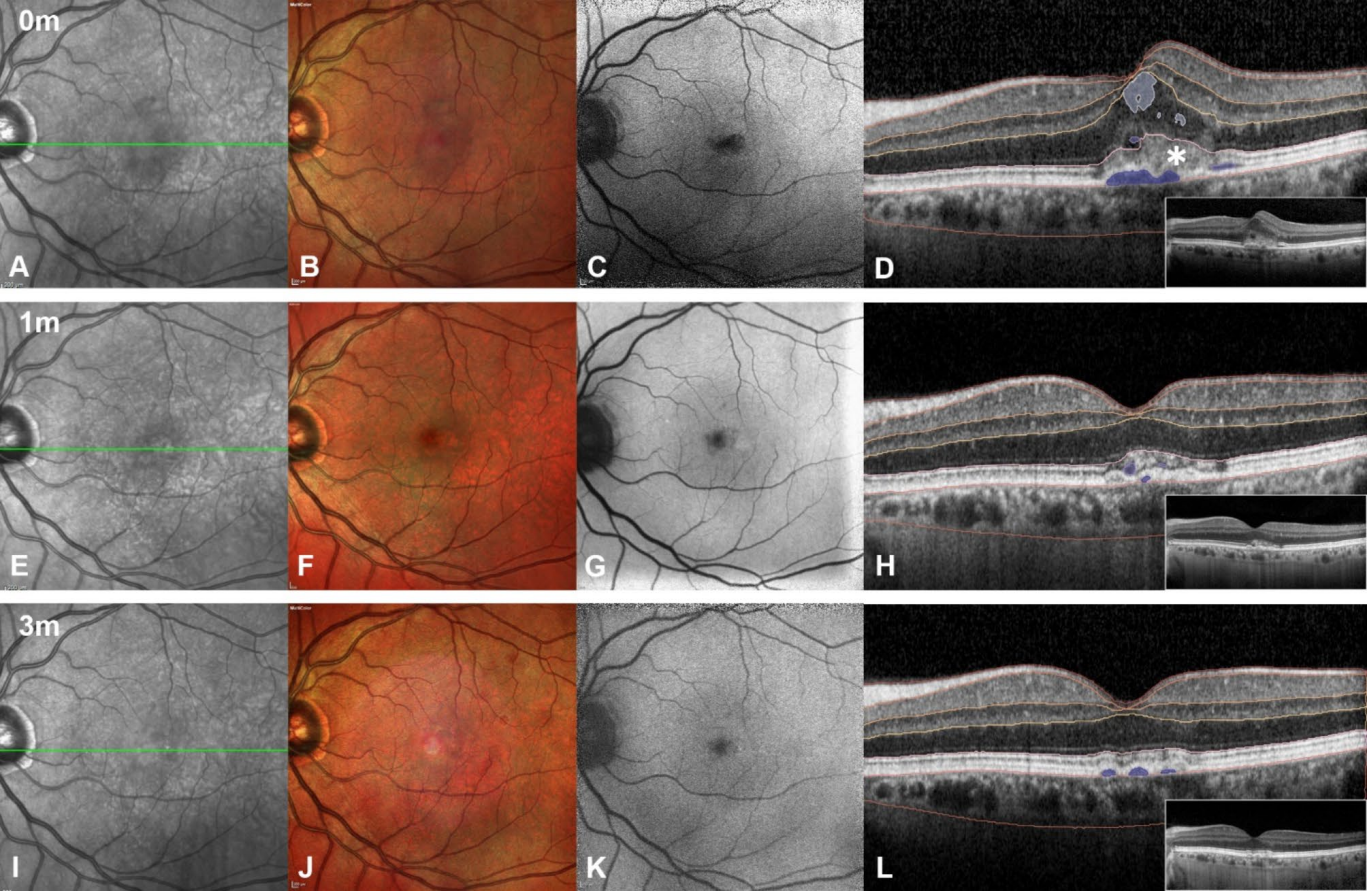
Patient example of the longitudinal changes in SRF, IRF, PED, TF volume. Multimodal imaging and fluid analysis obtained from the Discovery^®^ platform (RetinAI Discovery Layer and Fluid Segmentation and Biomarker Processor, Ikerian AG, Switzerland). (**A**-**E**-**I**) Infrared image respectively at baseline 1-month and 3-month follow up. (**B**-**F**-**J**) Multicolor imaging respectively at baseline 1-month and 3-month follow up. (**C**-**G**-**K**) Autofluorescence, respectively at baseline 1-month and 3-month follow up. (**D**-**H**-**L**) OCT scan segmented with AI software RetinAI (segmentation in violet indicates PED, light violet SRF, light blue IRF), respectively at baseline 1-month and 3-month follow up showing the early drying effect after IVT aflibercept 8 mg loading dose. SHRM (white star in **D**) resolution over the 3-month follow up can be observed

### Safety

A single ocular adverse event was observed during the observational period. The case involved a patient who received two monthly injections of aflibercept 8 mg. Three days after the second injection, the patient reported blurred vision without pain or redness. Slit-lamp examination revealed mild anterior chamber inflammation (1 + cells) in accordance with the SUN criteria [12]. Dilated fundus examination showed a clear vitreous without showing any evidence of retinal or vascular abnormalities on fundus examination. Topical treatment was initiated with prednisolone 1% eye drops, administered six times daily. After one week the inflammation gradually subsided. Steroid therapy was tapered over 30 days, leading to the complete resolution of inflammation without any vision loss. The patient was subsequently transitioned to an alternative anti-VEGF therapy, with no further adverse events. No additional ocular or systemic adverse events were observed in this study.

## Discussion

This study presents a short-term, real-world clinical experience with intravitreal aflibercept 8 mg in the management of patients with nAMD. We specifically focused on fluid volume control, as retinal fluid is a critical biomarker of disease activity, making its management a central objective in the treatment of nAMD [13]. In the registration trials, aflibercept 8 mg showed superior fluid control and non-inferior visual outcomes compared to aflibercept 2 mg, with these benefits maintained up to week 96. Our findings support the high efficacy of aflibercept 8 mg in achieving improved anatomical outcomes in nAMD patients, particularly by reducing total fluids [13]. To the best of our knowledge, this is the first study on aflibercept 8 mg utilizing AI-driven analysis to directly assess the efficacy of the molecule by quantitatively measuring fluid volume. This approach offers a novel perspective, as it enables precise quantification of fluid volumes through AI-based analysis software, further enhancing our understanding of treatment outcomes. Our findings indicate that aflibercept 8 mg demonstrated significant efficacy in control of exudation in nAMD as early as after the first injection (Fig. 2). Notably, a significant reduction in TF volume, in particular in SRF volume, was observed at 1-month and maintained at the 3-month follow-up. Additionally, the presence of SHRM [14], another feature of exudation, decreased after the 1st aflibercept 8 mg administration from 60 to 40%. This could indicate that the increased molar dose enhances the drying effect, which is sustained throughout the analyzed period. Increasing the molar dose of anti-VEGF agents has been investigated in the past as a potential strategy to optimize anatomical and clinical outcomes in the management of nAMD. Previous studies have shown that increasing the dosage of anti-VEGF agents can result in improved anatomical outcomes [15] and greater durability of treatment [16]. To explore these possibilities further, aflibercept 8 mg, was specifically engineered with enhanced solubility, viscosity, stability, and tolerability to optimize its pharmacodynamic profile. The phase 2 cd trial assessed the safety and efficacy of this higher dose in nAMD treatment-naive patients. These advancements emphasize the importance of ongoing research to determine whether higher molar doses can achieve superior anatomical and functional outcomes while extending treatment intervals. Our study showed that increasing the molar dose of aflibercept has demonstrated potential benefits and influenced the treatment approach, with only a subset of patients requiring a loading dose. Based on our real-world experience, the new aflibercept formulation appears to be an effective treatment option, demonstrating rapid fluid control and significant vision gains in a short term follow up. However, it is important to note that this study is exploratory and further studies with a longer follow-up are needed to evaluate the concept of “sustained disease control” over an extended period, to better understand whether vision gains, rapid and stable fluid control, and extended treatment intervals can be maintained over time [17]. Despite the higher volume of drug administered, no pressure spikes were observed, consistent with findings reported in the registration trials.

Regarding safety in our cohort, we reported one adverse event of mild anterior sterile intraocular inflammation. The inflammation was promptly diagnosed and effectively managed with topical corticosteroids, resulting in a complete resolution without any further sequelae. Recent literature has focused on sterile intraocular inflammation after intravitreal anti-VEGF agents. Montesel et al. described, in a large series of 10.297 injections of faricimab, a low incidence of IOI (0.19% per injection) with favorable prognosis [18]. However Cozzi et al. [19] reported the occurrence of rare but potentially serious IOI associated with faricimab. Regarding aflibercept 8 mg, the literature is still limited, with one publication by Hoffmann et al. [20], illustrating a cluster of 8 patients manifesting IOI after intravitreal aflibercept 8 mg at three different centers. IOIs were confined to the anterior chamber and the anterior vitreous and resolved with favorable visual outcomes following the initiation of topical steroid therapy, like our experience. Only 1 study in literature reported 3 cases (8,6%) of retinal vasculitis post aflibercept 8 mg injection [21]. The precise mechanism of IOI remains uncertain but may involve type III hypersensitivity reactions triggered by anti-drug antibodies or vascular endothelial cell damage caused by potent VEGF inhibition [22, 23]. There is also concern that high-dose anti-VEGF could increase the risk of IOI events. Therefore, we emphasize the importance of proactive screening to monitor for potential acute IOI episodes during follow-up visits after any type of intravitreal injection, including aflibercept 8 mg, to ensure overall treatment safety.

Our study highlights also the significant advantages of incorporating AI-driven analysis in evaluating intravitreal anti-VEGF treatment responses in nAMD. By enabling precise quantification of exudation volumes, AI provides insights that go beyond those achievable with traditional methods [24]. In recent scientific literature, Erfurth et al. employed a deep learning algorithm to detect and measure retinal exudations, including SRF, IRF, and PED, while also exploring the relationship between fluid volumes and visual function in AMD patients [25]. Consistent with previous research, our work also emphasized the importance of detecting and calculating fluid volumes for an accurate assessment of treatment response.

Limitations of our study included its retrospective design, a small sample size, and short follow-up period. Larger-scale, prospective, real-world studies with extended follow-up are needed to further evaluate the efficacy of aflibercept 8 mg in nAMD. Ongoing real-world studies will be essential for further assessing its efficacy, long-term durability and safety in daily clinical practice and to clarify the need of a loading doses. Moreover, the integration of AI-based analysis could play a key role in predicting treatment responses in larger datasets. AI-driven techniques offer the potential to refine real-world data analysis by identifying treatment outcome patterns, improving patient stratification, and facilitating personalized therapeutic strategies. Future studies with longer follow-up and AI applications will be crucial in assessing the role of AI in real-world clinical practice, particularly in standardizing treatment protocols and enhancing clinical decision-making.

## Conclusions

In summary, this study supports the effectiveness of intravitreal aflibercept 8 mg in the early control of retinal fluid volumes, reinforcing its importance as a valuable treatment option for managing vision-threatening conditions. Additionally, the use of the AI-based analysis allowed for detailed and automated assessment of retinal volume changes, providing valuable insights into early treatment effects. As an exploratory study, the results presented here should be interpreted as hypothesis-generating. Further studies are warranted to assess long-term outcomes and the potential predictive value of early changes on long-term fluid control and safety.

## Data Availability

No datasets were generated or analysed during the current study.

## Abbreviations

anti-VEGF: Anti-vascular endothelial growth factor
BCVA: Best-corrected visual acuity
CST: Central subfield thickness
ETDRS: Early treatment of diabetic retinopathy study
IOI: Intraocular inflammation
IOP: Intraocular pressure
IRF: Intraretinal fluid
IVT: Intravitreal injection
nAMD: Neovascular age-related macular degeneration
OCT: Optical coherence tomography
PED: Pigment epithelium detachment
SD: Standard deviation
SHRM: Subretinal hyper-reflective material
SRF: Subretinal fluid
RT: Retinal thickness
TF: Total fluid
T&E: Treat-and-extend
